# Deep neural learning based optimization for automated high performance antenna designs

**DOI:** 10.1038/s41598-022-20941-x

**Published:** 2022-10-07

**Authors:** Farzad Mir, Lida Kouhalvandi, Ladislau Matekovits

**Affiliations:** 1 Department of Electrical and Computer Engineering, University of Houston, 77204 Houston, Texas, USA; 2 Department of Electrical and Electronics Engineering, Dogus University, 34775 Istanbul, Turkey; 3 Department of Electronics and Telecommunications, Politecnico di Torino, 10129 Turin, Italy; 4 Faculty of Electronics and Telecommunications, Politehnica University Timişoara, 300006 Timişoara, Romania; 5 Istituto di Elettronica e di Ingegneria dell’Informazione e delle Telecomunicazioni, National Research Council of Italy, 10129 Torino, Italy

**Keywords:** Electrical and electronic engineering, Computational science

## Abstract

The present paper introduces an optimization-oriented method here practiced for designing high performance single antennas in a fully automated environment. The proposed method comprises two sequential major steps. The first one devotes configuring the shape of antenna and determining the feeding point by employing the bottom-up optimization (BUO) method. In this algorithm, the number of microstrip transmission lines (TLs) used to model the radiator is increased consecutively and the shape of the antenna is revised up to finding the initial satisfying results. Secondly, for determining the best design parameters of the configured antenna shape in the first step (i.e., width and length of TLs), deep neural network (DNN) that is based on Thompson sampling efficient multi-objective optimization (TSEMO) is applied. The recommended optimization method is successfully attracted as a problem solver for designers to tackle the subject for antenna design such as the complexity and large dimensions of structures. Hence, the main advantage of the implemented optimization method in this article is to noticeably decrease the required designer’s involvement automatically generating valid layouts. For validating the suggested method, two wideband antennas are designed, prototyped and subjected to experiment. The first optimized antenna covers the frequency band 8.8–10.1 GHz (13.75 % bandwidth) characterized by a maximum gain of 7.13 dB while the second one covers the frequency band 11.3–13.16 GHz (15.2 %) which exhibits a maximum gain of 7.8 dB.

## Introduction

Antennas become an important part in any hi-tech content device. The technology development has increased the need of various types of antennas subject to different constrains set by various considerations extending from electromagnetic to thermal going through mechanical or space limits. Due to their remarkable advantages such as cost-effective, consolidated effortless fabrication process, reduce space occupation, conformable, satisfactory bandwidth (BW), and adequate gain performance, microstrip patch antennas represent the most favorable class of antennas which are mostly used in communication systems^1^. As world population enjoys a noticeable rise, that in turn increases the need of mobile and hand held devices, improvement of antenna performances has been sensed in order to provide the appropriate BW and nearly flat and constant gain performance in the operational frequency band(s)^2^.

One of the major critical design challenges consists of fulfilling the design goals in the considered operation BW. Hence, designing and optimizing antennas considering conventional electronic design automation (EDA) tools for achieving proper BW and very nearly constant gain would be rather difficult due to the complexity of antenna structures. During the last decade, a variety of optimization methods have absorbed the attention of designers to tackle the drawbacks of EDA tools in this sense. Some of the various reported optimization methods are: surrogate-based optimization^3^, particle swarm optimization^4^, spider monkey optimization^5^, genetic algorithm optimization^6,7^, and K-nearest neighbor algorithm^8^. These algorithms are successful set of rules; however, due to the complexity of antennas more accurate and advanced multi-objective optimization techniques are required for optimizing designs. Recently, neural networks have proved their reliable performance and competence in modeling high dimensional radio frequency (RF) designs^9–14^. Deep neural networks (DNNs) in opposite to the shallow neural networks (SNNs), include multi layers in their structures that results in more accurate performance in the complex and high-dimensional designs^9^.

To the best of authors’ knowledge, it is for a very first time in the literature where a fully automated optimization method is presented for configuring the antenna’s structure and sizing the design parameters, respectively. The automated algorithm includes two main optimization procedures. The first step optimization is for configuring the initial antenna shape using the bottom-up optimization (BUO) method^15^ where the number of microstrip transmission lines (TLs) are increasing sequentially and the suitable feeding point is optimized. And the second step of optimization devotes to sizing the antennas and augmenting antenna performances using the DNN-based Thompson sampling efficient multi-objective optimization (TSEMO) algorithm^16^. This algorithm is selected in this work due to the successful reductions in the hypervolume calculations^16^. The regression DNN is applied for accurately modeling and enhancing the overall performance and is devoted to synthesizing the final post-layout of antenna by applying electromagnetic (EM)-verified fabrication rules.

The present paper is organized as follow: in “Details of the proposed method” the proposed optimization method is presented. Section “Practical implementation of proposed automated optimization strategy” is devoted to the implementation of the proposed automated-optimization strategy. Section “Fabrication and measurement” describes the practical antenna design and verification by applying the proposed method. Fabrication details and measurement results are provided in this section. Finally, conclusions are presented in “Conclusion”.

## Details of the proposed method

This section provides brief details of the two employed optimization methods for modeling the antenna structure and sizing the design parameters of the configured antenna, respectively. The design goals are $S_{11}$, and gain specifications at the whole frequency band that are achieved by sizing the design parameters using our proposed method.Constructing the initial antenna shape and optimizing the feeding point are performed by using the BUO method while sizing the antenna parameters is achieved with the multi-layer neural network (i.e., DNN) using multi-objective optimization method. Here, the theories of these optimization methods are described clearly. Whole of the optimization process is automatically executed that results in high performance single antenna designs in terms of flat gain and wide BW. Following is the in detail descriptions of proposed method.

### Optimizing the initial antenna configuration

**Figure 1:**
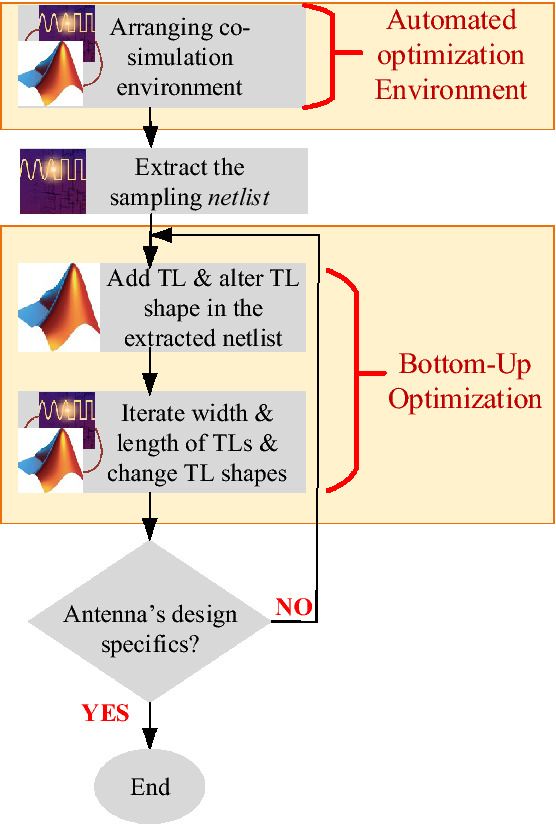
Flowchart of employed BUO method for primarily configuring the antenna shape.

Determining the initial antenna shape is critical which paves the way of designers. For this case, the BUO method is employed for configuring the primary antenna shape and also for determining the suitable feeding point. In this part, we explain the theory of BUO method in detail which automatically generates the initial antenna geometry with feeding point. The summary of employed BUO method is provided in Algorithm 1 and Fig. 1 at the end of this section.

#### Constructing an automated environment

For minimizing the designers’ interruptions, fully automated optimization environment is created by the combination of EDA tool such as keysight ADS and the numerical analyzer as MATLAB. In this phase, the ADS software works in the background and the MATLAB tool handles the generated output data from the file namely as *spectra.raw*^17^ and does the mathematical analysis.

#### Employment of the BUO method

The BUO method is a combination of lower-level components where the higher-level design is decomposed into several hierarchical blocks similarly as it is done in a general domain decomposition^15^. Providing the starting point of optimization that represents the general geometry of the single antenna is a significant role. Hence, this optimization method is employed where both the single antenna configuration with feeding point are determined and optimized. The BUO algorithm considers the fine-tuning exploiting the sensitivity of the resonance to the geometrical dimensions. With the help of this method, the initial configuration of antenna is generated through BUO method that the number of transmission lines are increasing sequentially. All the data (including the width and length of transmission lines) is saved in the output file namely as ‘spectra.raw’ file.

As presented in “Constructing an automated environment”, firstly the automated environment that is the combination of ADS and MATLAB is created. Afterwards a sample *netlist* file is created from ADS and then the optimization process is initialized. In this algorithm, the number of TLs is increasing continuously and various shape of TLs are examined up to achieving primary responses in terms of gain and band frequency. Beside, the suitable biasing point is also determined by the help of this algorithm. The output responses are achieved in the spectra.raw file and MATLAB is analyzing these generated outcomes. 
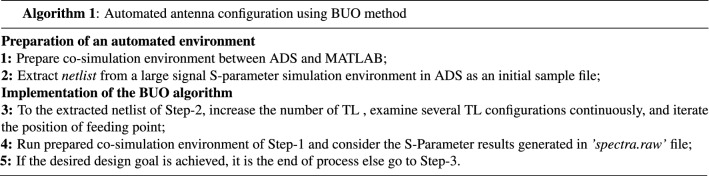


### Predicting and sizing the antenna parameters using DNN

After determining the suitable antenna configuration, it is time to obtain the optimized design parameters as length (L) and width (W) of included TLs. The target of this part is to enhance the flatness of ^16^gain within the BW. Hence, advanced multi-objective optimization methods are required to deal with multi-objective specifications. In this work, we employ the TSEMO algorithm by using the regression DNN. The TSEMO algorithm is selected in this works due to the fast optimization process by building the Gaussian process surrogate models and also the ability to evaluate different simulations, in parallel. For applying the TSEMO algorithm the DNN, includes multi-layers and optimal neurons, is used which results in accurate antenna modeling. In this section, firstly the theory of the TSEMO method is explained and then the procedures for constructing and using the DNN in the multi-objective optimization is described, briefly.

#### Thompson sampling efficient multi-objective optimization (TSEMO)

##### TSEMO inference

After constructing the initial configuration of the single antenna, the optimized values for design parameters must be achieved. The brief definitions for this algorithm is as following:

The TSEMO algorithm as a multi-objective optimization is defined in (1):

1 $$\begin{aligned} \mathrm{minimize}_{x \varepsilon \chi \subseteq {\mathbb {R}} ^d } G(x) = \begin{bmatrix}g_{1}(x),g_{2}(x),\ldots ,g_{m}(x)\end{bmatrix} \end{aligned}$$

where $\chi$ is the design space, *x* is the decision vector and *G* is a vector of *m* objective functions ($g_{i}$*(x)*).

**Figure 2:**
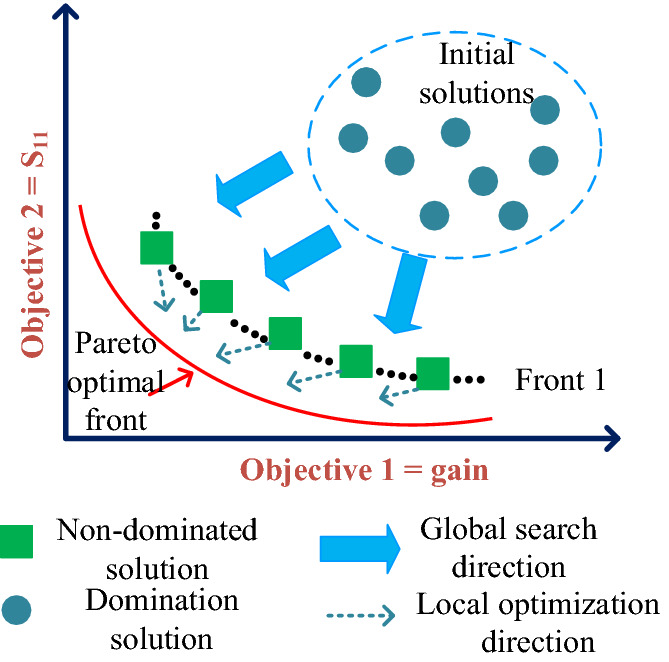
Example of Pareto front in two-objective optimization.

Generally, this algorithm is used in global multi-objective optimization of expensive-to-expensive black-box functions. It is based on Bayesian optimization (BO) approach that builds Gaussian process (GP). The single objective method of TSEMO in BO is to find the accurate global minimizer $x^{*}$ of a function *g*($x^{*} \varepsilon argmin_{x \varepsilon \chi \subseteq {\mathbb {R}} ^d } g(x)$). This single-objective in BO is extended to the multi-objective case. Basically, TSEMO is getting points to approximate the pareto optimal front (POF) of the different objective functions. Figure 2 shows an example of two objective functions that the final output includes the points that are close to the Pareto set.

##### TSEMO algorithm outline

To start the optimization, initial data set from space-filling design such as a Latin hypercube design^18^ is needed for initializing GPs. The detailed description of GP is described below up to (8). The prior Gaussian distributed noise with noise variance $\sigma _n ^{2}$ is as follows:

2 $$\begin{aligned} y(x) \sim GP(m(x),k(x,\acute{x})) \end{aligned}$$

with mean function (3) and covariance function (4), respectively.

3 $$\begin{aligned} m(x):= & {} {\mathbb {E}}_{f}[f(x)] \end{aligned}$$

4 $$\begin{aligned} k(x,\acute{x}):= & {} {\mathbb {E}}_{f}[(y(x)-m(x))(y(\acute{x})-m(\acute{x}))] \end{aligned}$$

The posterior definition of GP is needed as the prior of GP does not depend on observations. The refinement of prior by using Bayes’ rule is as follows:

5 $$\begin{aligned} f(x) \sim GP (m(x),k(x,\acute{x}) \mid X,Y) \end{aligned}$$

where

6 $$\begin{aligned} m(x) \mid X,Y= & {} \sum (x,X) \sum \nolimits _{}^{-1} y \end{aligned}$$

7 $$\begin{aligned} k(x,\acute{x})\mid X,Y= & {} k(x,\acute{x}) - \sum (x,y) \sum _{}^{-1} \sum (x,X)^T \end{aligned}$$

for *n* points of $$X= \begin{Bmatrix}x_{1},\ldots ,x_{n}\end{Bmatrix}$$ where $x_{i}$ is $$\begin{bmatrix}x_{i1},\ldots ,x_{id}\end{bmatrix} ^T$$ and $y_{i}$ is the corresponding observations at $x_{i}$ for the set $$Y= \begin{Bmatrix}y_{1},\ldots ,y_{n}\end{Bmatrix}$$ and the vector $$y= \begin{bmatrix}y_{i1},\ldots ,y_{id}\end{bmatrix} ^T$$.

Therefore, for training hyperparameters the maximum posterior estimate (MAP) for inferring is used. The MAP hyperestimation is given in (8) where ${\mathscr {L}}_{MAP}(\xi )$ is the MAP likelihood.

8 $$\begin{aligned} \xi _{MAP} \varepsilon argmax_{ \xi } {\mathscr {L}}_{MAP}(\xi ) \end{aligned}$$

Secondly, a set of candidate for sampling must be specified. The inputs of the data collected and the corresponding responses of each function $,g_{j}(x)$ with $j=1,\ldots ,m$ are $$X^{i} := \begin{Bmatrix}x_{1},\ldots ,x_{n},x_{n+1},\ldots ,x_{n+i+1}\end{Bmatrix}$$ and $$Y_j^{i} := \begin{Bmatrix}y_{j}^{1},\ldots ,y_{j}^{n},y_{j}^{n+1},\ldots ,y_{j}^{n+i}\end{Bmatrix}$$, respectively. For each $Y_j^{i}$ independent GP is trained as described before. In this case, *m* sample functions are obtained as $$\begin{Bmatrix}f_{1}^{i}(x),\ldots ,f_{m}^{i}(x)\end{Bmatrix}$$. The approximate Pareto set of the sampled functions at each iteration is found.

For the output data set as $$\begin{Bmatrix}Y_{1}^{i},\ldots Y_{m}^{i}\end{Bmatrix}$$ and current reference point for the hypervoume calculation defined as $\mathbf{r }^i$, Pareto front is defined as $\rho ^i$. In this optimization algorithm (i.e., TSEMO) the aim is to find the maximum hypervolume improvement ($\Delta HV$) added to $\rho ^i$ (Pareto front) (9).

9 $$\begin{aligned} x_{n+i+1}\in argmax HV(\rho ^i \cup \begin{Bmatrix}y_{c}\end{Bmatrix},\mathbf{r }^i ) - HV (\rho ^i ,\mathbf{r }^i) as \qquad \qquad x \in C^i \end{aligned}$$

Lastly, the data sets as $$X^{i+1} := \begin{Bmatrix}x_{1},\ldots ,x_{n},x_{n+1},\ldots ,x_{n+i+1}\end{Bmatrix}$$ and $$Y_j^{i} := \begin{Bmatrix}y_{j}^{1},\ldots ,y_{j}^{n},y_{j}^{n+1},g_{j}^{x_{n}+i+1}\end{Bmatrix}$$ for $j=1,\ldots ,m$ are updated and repeated up to obtaining the determined goal.

#### Construction of the regression DNN

**Figure 3:**
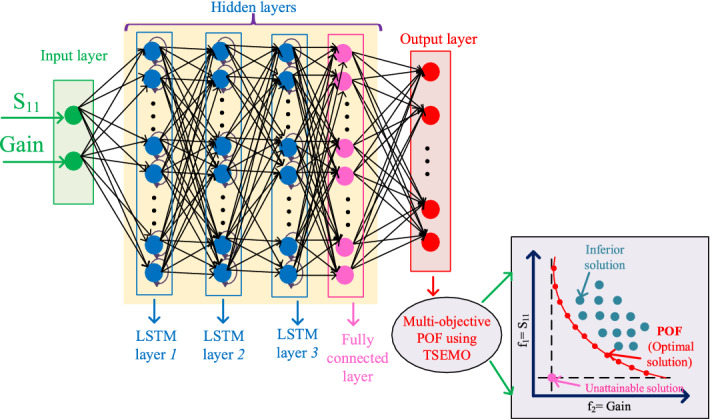
Proposed regression DNN for sizing the antenna shap with BUO.

For constructing an accurate regression DNN, some requirements must be set that are: suitable amount of data set, number of hidden layers with number of neurons, and also input and output layer features. Figure 3 presents the general structure of the regression DNN aiming to predict the optimal component values, automatically. The detail descriptions for each named requirement is as following:

##### Data generation

After configuring the initial antenna shape, optimal design parameters must be determined. All the optimization process is performed automatically in the created platform described in “Constructing an automated environment”. For accurately modeling the antenna, a suitable amount of data set includes training, validation and test data ($X_{Train}$, $X_{Val}$, and $X_{Test}$), and corresponding desired outputs ($Y_{Train}$, $Y_{Val}$, and $Y_{Test}$) sets are needed. The generated data set is split into three groups of training, validation, and testing data with the rates of 70%, 15%, 15%, respectively.

Any designer can select this ration by his/her idea. What is an important concept is to devote large ratio of data for training since as much as data is large, the neural network will be constructed accurately. The target of each set is as following:

- Training set: This set is used for training the model such as antenna and to encourage the model to learn the hidden features. In this case, the model is trained for all diverse data and will be able to predict the unseen future data. As the neural network is trained with these data, large ratio of data set (i.e., $70\%$) must be devoted to training the neural network.
- Validation set: The validation set is used to answer this question to the designer that: the model training is done in accurately or not. Hence, $15\%$ of data is devoted to this data set.
- Testing set: After completing and training the neural network, the last divided $15\%$ of data set is devoted to test that the trained neural network.

For the configured initial antenna shape in “Employment of the BUO method”, the related ’netlist’ file is extracted and then randomly the design parameters (include W and L of TLs) are changed within the different range of ${\mp }5\%$, ${\mp }10\%$, and ${\mp }15\%$. The large amount of simulation results for different parameters are presented in the output file namely ’spectra.raw’. Each of the output files consist of $S_{11}$, and gain results which are the input features for the depicted regression DNN in Fig. 3. For each achieved output response, the POF of two functions (i.e., $S_{11}$, and gain) is obtained using the TSEMO method (see Fig. 2). These POF responses yields the output layer’s parameters.

##### Structure of the DNN

**Figure 4:**
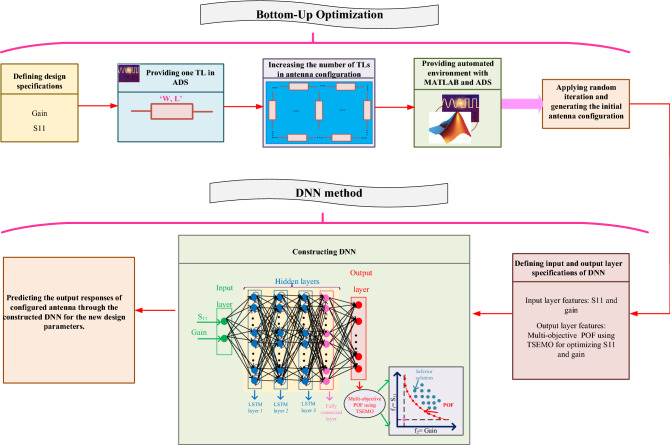
Flowchart of our proposed method.

As explained before, DNNs consist of multiple layers that each layer include some neurons. Determining optimal hyperparameters (i.e., number of hidden layers with neurons) is not straightforward and requires optimization techniques as well. In this work, we apply the BO for predicting the optimal hyperparameters of the employed regression DNN as it is more accurate than grid search and randomized search^19^. The employed DNN in this work consists of three *Long short-term memory (LSTM)* layers and one fully connected layer as Fig. 3.The used activation function and loss function are the rectified linear unit (ReLU) and the mean squared error, respectively. After constructing the DNN, this neural network refer to the ‘spectra.raw’ file includes the sizes of transmission lines and picks the values of gain and S11 from this file to employ the TSEMO method and finding the optimal design parameters. The overall flowchart of our proposed methodology is provided in Fig. 4.

A summary of the proposed algorithm for obtaining the best component values is the following: 
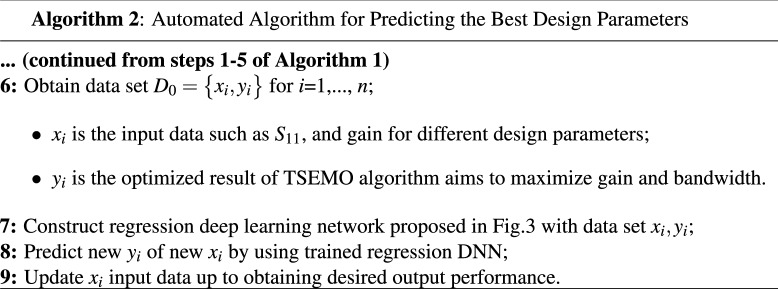


## Practical implementation of proposed automated optimization strategy

**Figure 5:**
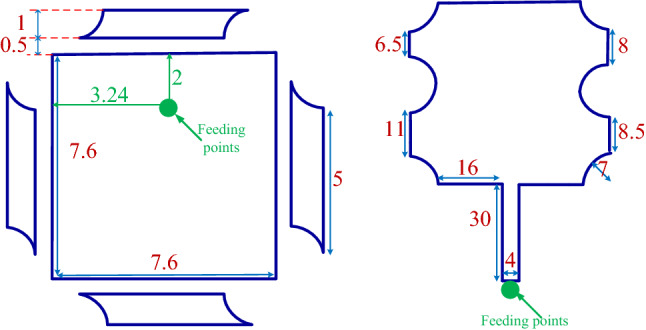
Initial configured antennas using the BUO method; antenna-1 (left) antenna-2 (right). Unit is mm.

**Figure 6:**
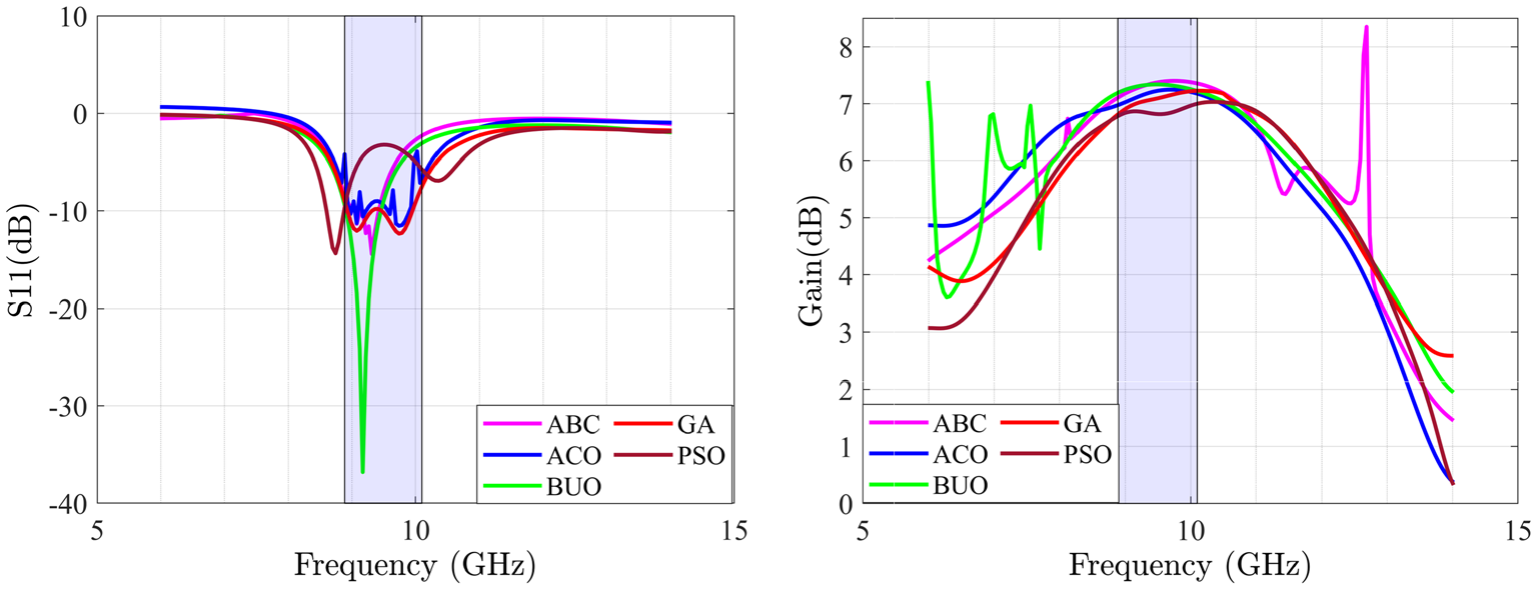
$S_{11}$ parameter of antenna-1 with ABC, ACO, BUO, GA and PSO methods (left); gain of antenna-1 with ABC, ACO, BUO, GA and PSO methods (right).

**Figure 7:**
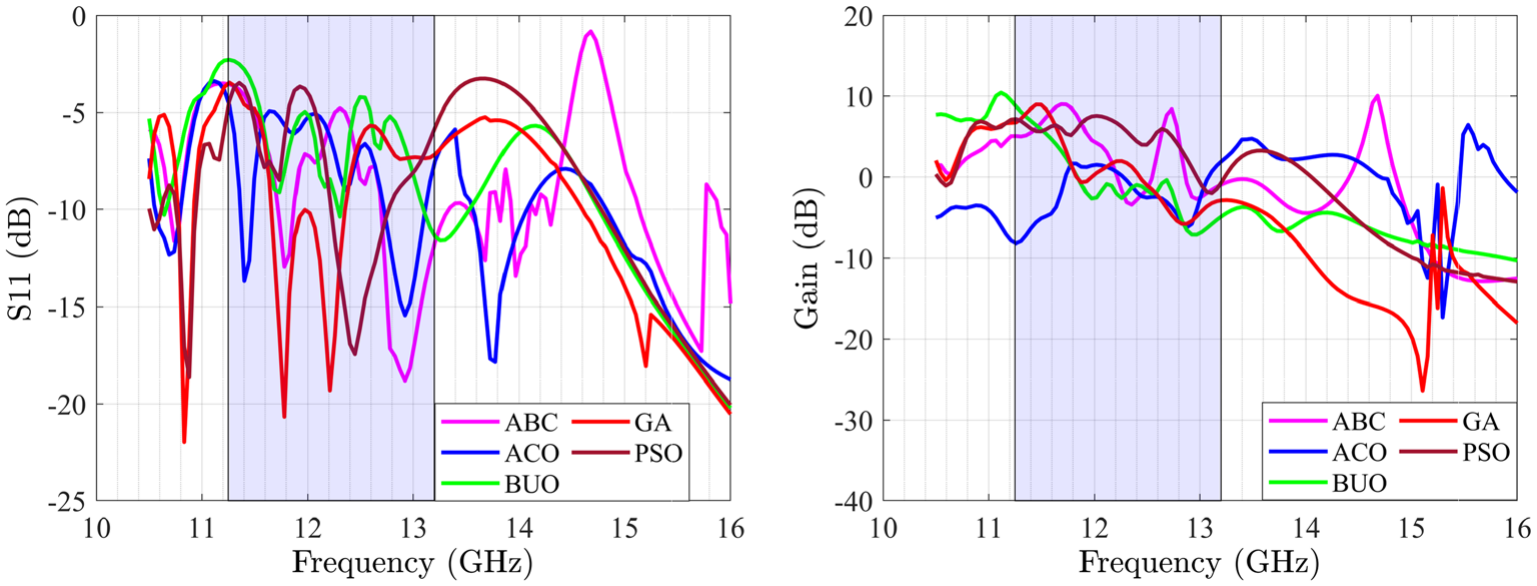
$S_{11}$ parameter of antenna-2 with ABC, ACO, BUO, GA and PSO methods (left); gain of antenna-2 with ABC, ACO, BUO, GA and PSO methods (right).

This section provides a sequential construction of the proposed optimization strategy that is performed automatically. In the first phase, the general antenna structure with location of the feeding point are determined using the BUO method. For this case, the BUO method is employed where Fig. 5 presents the two generated microstrip antennas where 12 design parameters for the first antennas and 13 design parameters for the second one are provided. The initial structure starts with a simple square block and by applying the BUO method, the structure of the antenna became updated and the final configuration is achieved. As these antennas are consisting of TLs, the constrains presented in^20^ are applied during the optimization process for passing the EM simulations successfully. As Figs. 6 and 7 illustrate, the BUO method is suitable enough for generating the initial configuration of antennas in comparison with genetic algorithm (GA), particle swarm optimization (PSO), ABC (Artificial Bee Colony), and ACO (Ant colony optimization). As it is clear, these algorithms could not be powerful for achieving high performance outcomes, so substantial need for the our proposed method in “Predicting and sizing the antenna parameters using DNN” is required.

In our proposed method, the LSTM layers are used for optimizing antennas in a large frequency band. In this method, gain and $S_{11}$ performances of each frequency with the bandwidth of 100 MHz are optimized. For this case, the proposed DNN in Fig. 3 is used which is based on the TSEMO algorithm. As described before, the employed DNN consists of three LSTM layers with one fully connected layer. By applying the BO method, 3 layers where each layer includes 250 neurons are determined where the normalized root mean square error (RMSE) for the constructed regression DNN becomes 0.10 and 0.12 for the first and second optimized antennas, respectively. In total for both antennas, 3500 sequences with divided data of 2450, 525, and 525 sequences for $X_{Train}$, ${X_Val}$, and $X_{Test}$, respectively. Each sequence includes multi-segment $S_{11}$ and gain values in the required band frequency.

## Fabrication and measurement

**Figure 8:**
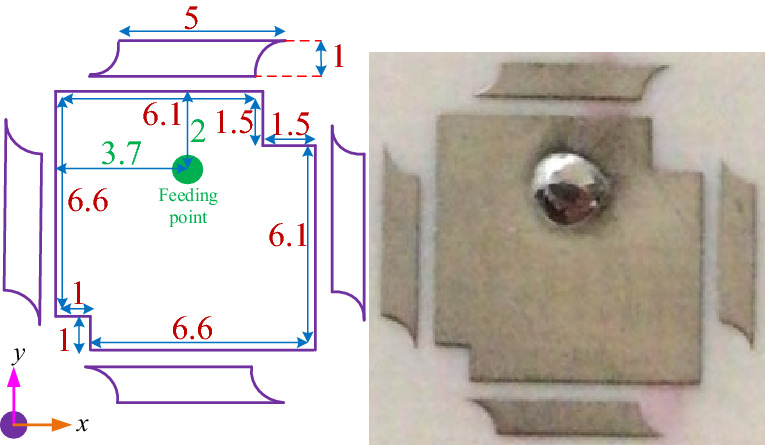
Optimized antenna-1; simulated (left) fabricated (right). Unit is mm.

To verify the proposed method of optimization, two microstrip patch antennas have been designed and fabricated as illustrated in Figs. 8 and 12. In this paper, two different antennas in terms of their structures have been fabricated on Rogers 4003 substrate with $\tan \delta = 0.0027$, $\varepsilon _r = 3.55$ and thickness of 1.52 mm. For both antennas, the final configurations with feeding point are achieved using BUO, and the design parameters are predicted automatically using the DNN that is based on the TSEMO method.

**Figure 9:**
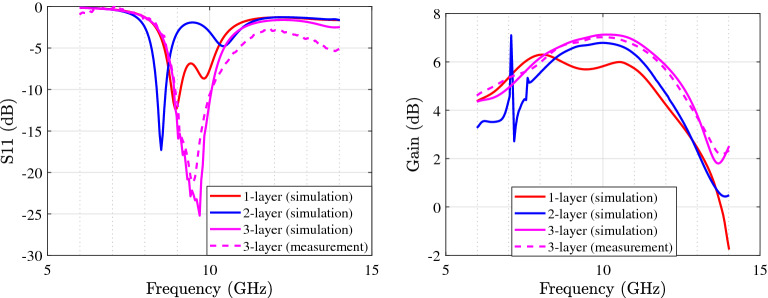
$S_{11}$ parameter of antenna-1 (left); gain of antenna-1 (right).

**Figure 10:**
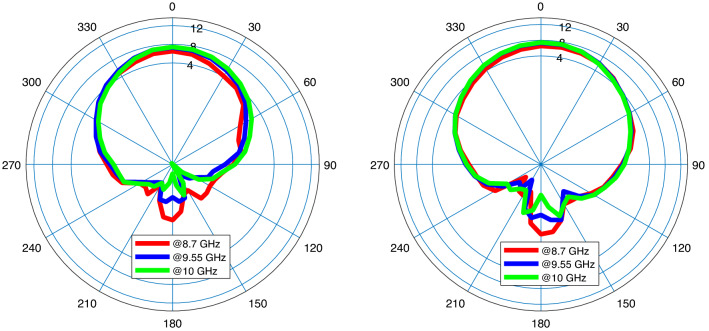
Radiation pattern of simulation antenna-1 at $f_{1}=8.7$ GHz (red), $f_{2}=9.55$ GHz (blue), and $f_{3}=10$ GHz (green); $\phi =0$ (left), $\phi =90^{\circ }$ (right).

**Figure 11:**
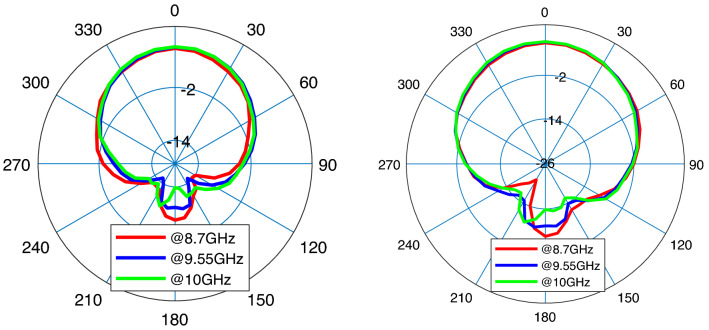
Radiation pattern of fabricated antenna-1 at $f_{1}=8.7$ GHz (red), $f_{2}=9.55$ GHz (blue), and $f_{3}=10$ GHz (green); $\phi =0$ (left), $\phi =90^{\circ }$ (right).

Figure 8 presents the first microstrip patch antenna which is designed by using two triangular shapes on the top right and the down left of the structure. In this single antenna, the final design parameters have been prepared that are estimated using the proposed DNN. This antenna exhibits a 32.5 % impedance BW which covers 8.8–10.1 GHZ as it is shown in Fig. 9 (left). Our antennas are designed to work in X and K frequency bands which are mainly used for communication and satellite functions (radar signals). These frequency bands are selected as in our future work, we will use these antennas for matching to the working frequency band of aimed power amplifiers. For clearly observing the effects of more hidden layers and the more accuracy output response in the third layer during the optimization process, the performance of $S_{11}$ and gain are presented with three hidden layers. Any neural network can be either shallow neural network (i.e., a network with one hidden layer) or deep neural network (i.e., a network with more than one hidden layer). We start optimizing antenna with a neural network with one hidden layer and we develop the number of layers. When the desired output specifications are achieved, increasing the hidden layers are stopped. So in our simulation environment, the automated process is stopped when the desired specifications are obtained. For our antennas, when the number of hidden layers becomes 3, the optimization process is stopped that is meaning that the desired antenna goals are obtained. For the mentioned impedance BW of the optimized antenna, the gain performance is shown in Fig. 9 (right) and it is between 6.8 and 7.13 dB which represents the flat gain for this antenna. The minimum return loss for antenna-1 is $-22$ dB which takes place at 9.7 GHz. By achieving the optimized design parameters from the optimization method, this antenna has been fabricated that both impedance BW and gain performance depict a good matching as illustrated in Fig. 9. As it is represented in Figs. 10 and 11 radiation pattern for both simulated and measured antenna-1 at three different frequencies 8.7 GHz, 9.55 GHz and 10 GHz have been achieved according to the value of $\phi = 90$ and $\phi = 0$, respectively.

**Figure 12:**
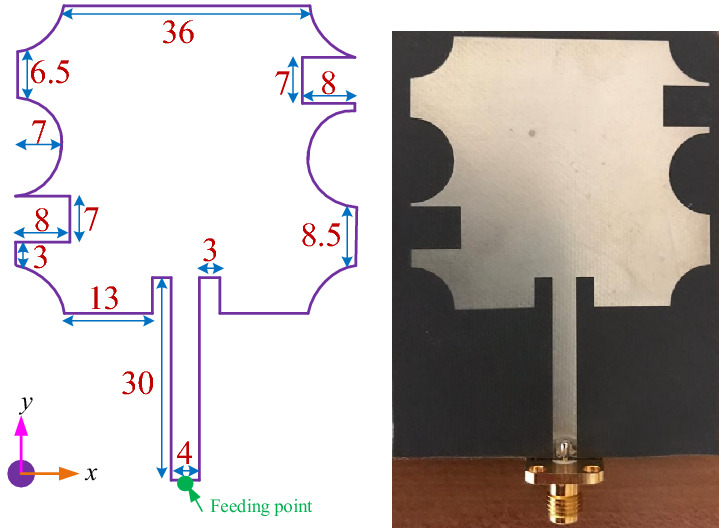
Optimized antenna-2 simulated (left) fabricated (right). Unit is mm.

For validating the proposed optimization strategy, another single microstrip antenna is optimized. Figure 12 shows the optimized antenna shape with the appropriate dimensions. In this antenna, more efforts have been done such as two circle with 7 mm radius at the both side of the antenna, also four circle with the same radius as above hinted are provided at the corners of the antenna.

**Figure 13:**
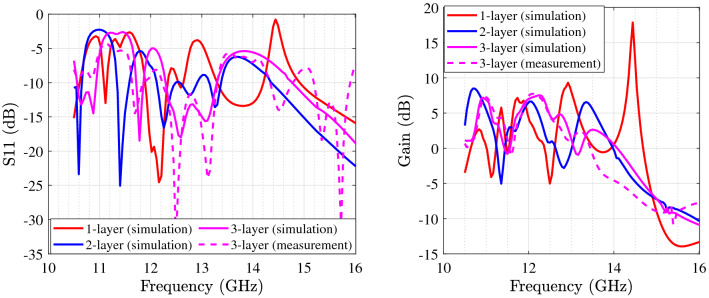
$S_{11}$ parameter of antenna-2 (left); gain of antenna-2 (right).

**Figure 14:**
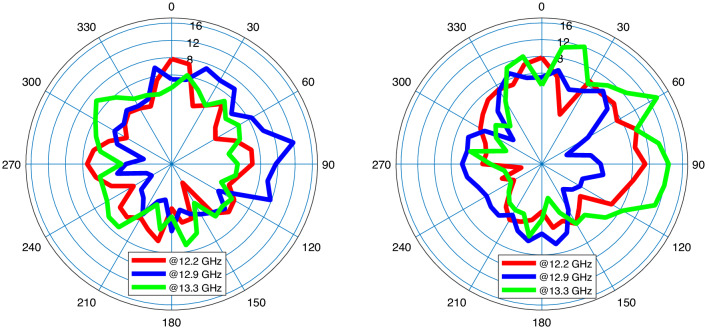
Radiation pattern of simulation antenna-2 at $f_{1}=12.2$ GHz (red), $f_{2}=12.9$ GHz (blue), and $f_{3}=13.3$ GHz (green); $\phi =0$ (left), $\phi =90^{\circ }$ (right).

**Figure 15:**
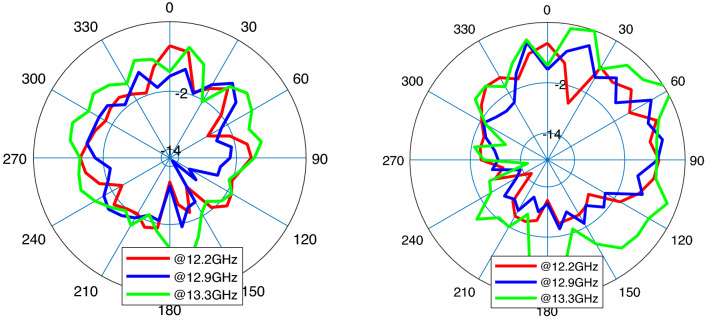
Radiation pattern of fabricated antenna-2 at $f_{1}=12.2$ GHz (red), $f_{2}=12.9$ GHz (blue), and $f_{3}=13.3$ GHz (green); $\phi =0$ (left), $\phi =90^{\circ }$ (right).

Figure 12 depicts the final and optimized design parameters which covers the frequency band from 11.3 to 13.16 GHz (37.2 % of total range usable bandwidth). Figure 13 (left) represents $S_{11}$ for different hidden layers. Gain performance for this type of antenna shows an acceptable gain and reaches to 7.8 dB in the third layer as expressed in Fig. 13 (right). The final design parameters has been approached after analyzing the antenna with three hidden layers. Thus, the antenna with finalized design parameters has been fabricated with the same process. Figure 13 illustrates a perfect matching for both simulated and measured antenna. The minimum $S_{11}$ ($-18$ dB) for antenna-2 which happens at 11.7 GHz. The same process of radiation pattern in Figs. 14 and 15 have been implemented for simulated and measured antenna-2 for its radiation pattern which has been tested at three different frequencies as hinted above for two value of $\phi =90$ and $\phi = 0$. The overall time costs for generating the initial antenna configurations and sizing the design parameters for antenna-1 and antenna-2 are around 3 h 30 min and 3 h 50 min, respectively. Hence, any designer can get benefit of this method for automatically generating post-layouts without manual interruptions and with reduced attempt.

## Conclusion

The purpose of this study is to apply an automated optimization method by the means of the BUO and DNN based multi-objective optimization to obtain desired BW and flat gain performance. Proposed optimization methodology in this paper is presented for the very first time in the literature and includes two phases, which are applied sequentially. The first phase is responsible for configuring the initial single antenna configuration and also for determining the suitable feeding point. The second step of the optimization corresponds to applying the multi-objective algorithm as TSEMO method through regression DNN to determine the design values, that in turn gives rise to achieve the design goals. The regression DNN helps designers to model the complex antenna designs accurately where it provides a regression environment for applying multi-objective algorithms. The advantage of our proposed method is to reduce the designer intervention and prepare ready-to-fabrication layouts by applying fabrication rules and constrains. To verify the effectiveness of the proposed method, two single microstrip antennas in the frequency band of 8.8–10.1 GHz and 11.3–13.16 GHz are designed, fabricated, and measured where the measurement results show well-agreement convergence to the simulated results. In our method, two specifications as gain and $S_{11}$ are optimized and broadside direction with radiation efficiency will be considered as the future work.
