# Author Correction: Deep neural learning based optimization for automated high performance antenna designs

**DOI:** 10.1038/s41598-022-23916-0

**Published:** 2022-11-10

**Authors:** Farzad Mir, Lida Kouhalvandi, Ladislau Matekovits

**Affiliations:** 1 Department of Electrical and Computer Engineering, University of Houston, Houston, TX 77204 USA; 2 Department of Electrical and Electronics Engineering, Dogus University, 34775 Istanbul, Turkey; 3 Department of Electronics and Telecommunications, Politecnico di Torino, 10129 Turin, Italy; 4 Faculty of Electronics and Telecommunications, Politehnica University Timişoara, 300006 Timişoara, Romania; 5 Istituto di Elettronica e di Ingegneria dell’Informazione e delle Telecomunicazioni, National Research Council of Italy, 10129 Turin, Italy

Correction to: *Scientific Reports* https://doi.org/10.1038/s41598-022-20941-x, published online 07 October 2022

The original version of this Article contained errors in the Abstract.

“The first optimized antenna covers the frequency band 8.8–10.1 GHz (43% bandwidth) characterized by a maximum gain of 7.13 dB while the second one covers the frequency band 11.3–13.16 GHz (47.5%) which exhibits a maximum gain of 7.8 dB.”

now reads:

“The first optimized antenna covers the frequency band 8.8–10.1 GHz (13.75% bandwidth) characterized by a maximum gain of 7.13 dB while the second one covers the frequency band 11.3–13.16 GHz (15.2%) which exhibits a maximum gain of 7.8 dB.”

The original Article has been corrected.

